# The Dialectical Mandala Model of Self-cultivation

**DOI:** 10.3389/fpsyg.2022.1024413

**Published:** 2023-01-25

**Authors:** Orchid-Stone Chang Azanlansh

**Affiliations:** Undergraduate Degree Program of Rift Valley Interdisciplinary Shuyuan, College of Huilan, National Dong Hwa University, Shoufeng, Taiwan

**Keywords:** cross-cultural, dialectic, mandala, ontological model, self-cultivation

## Abstract

This study explores the development of a cross-cultural primary ontological model that can help self-cultivation practitioners illuminate their path and help researchers identify the complex implications, context, and progression of self-cultivation in diverse cultures, especially those associated with Buddhism, Taoism, and Confucianism. Integrating self-cultivation traditions into social science research from the perspective of subject-object dichotomy is difficult. However, the assimilation of the mutual implication of subject and object in the Avataṃsaka worldview helps resolve this issue. This study employs the Buddhist tetralemmic dialectic (catuṣkoṭi), which goes beyond the limitations of dualistic and reductionist logic, to construct the Dialectical Mandala Model of Self-cultivation as the first of a two-step epistemological strategy. The model provides a universal framework for the multifaceted and systemic analysis of self-cultivation traditions so that future research can further develop additional culturally specific ontologies and psychological models in the second step of the strategy. As in a research map, this model could help researchers make ontological commitments, understand self-cultivation more comprehensively, and determine whether they have overlooked any research domain.

## 1. Introduction

### 1.1. Self-cultivation as a cross-cultural phenomenon

Self-cultivation is the development and integration of mind and body through self-effort; it aims to develop one’s potential, integrate experiences and awareness, reach beyond primitive states of being (Tang, 2015), and, from the Western perspective, attain transcendence (Peters, 2020). Concepts and practices of self-cultivation are found in many cultures, but they are accomplished through diverse processes and worldviews in each (Hwang and Chang, 2009). For example, the German idea of self-cultivation (*Bildung*) can be historically traced to Hegel and is defined as a disciplined effort directed at spiritual self-development (Gadamer, 2004; Peters, 2020). By contrast, self-cultivation, as an essential component of established East Asian ethical values, reflects an inherent tendency toward harmony and wholeness, which culminates in a more natural state beyond the self (Tang, 2015; Shiah, 2016; Yü, 2016).

Although many traditions use the term, the related concepts significantly differ in each tradition. For example, the classical idea of transcendence in Greek-inspired traditions—in which the object and goal (e.g., God and heaven) are independent of the individual—has no counterpart in certain Eastern traditions such as Zen Buddhism. Accordingly, some Zen masters, such as Mazu Daoyi (709–788) and Xuefeng Yicun (822–908), posit internal “ghosts” rather than an external God to help their disciples work on self-cultivation (*Taishō Tripiṭaka* [hereafter “T.”] 51, p. 351c, ll. 19–20); therefore, they emphasize “ordinary mind” and “no transcendence” (Magid, 2012, p. 86; T. 51, p. 440a, ll. 3–6). For Zen masters, self-cultivation is not about changing things or denying the self; it is about leaving things as they are, as long as the mind is no longer obscured by presuppositions about the self (Sanskrit [Skt.] *ātma-grāha*). Analogously, Yü (2016) interprets the kernel of self-cultivation as “inward transcendence” because self-cultivation in Chinese traditions is grounded in the holistic concept that the world and the self are one (p. 16). Such ontological differences in concept and practice have barely been addressed in cross-cultural research.

### 1.2. The dilemma of cross-cultural research: The need to develop ontological models

Cross-cultural research on self-cultivation and spirituality is faced with a fundamental dilemma: psychological variables are observed and measured under different cultural conditions, sometimes without an ontological understanding of these variables (Genkova, 2015). Using identical measuring instruments (e.g., questionnaires) and procedures does not guarantee measurement equivalence, because both the stimuli (e.g., the questions) and the responses may have different meanings in diverse cultures (Byrne and Watkins, 2003). For example, Hofstede (2011) identifies six dimensions that differentiate one culture from another. One of these is individualism versus collectivism, referring to how one relates to others within the same community. Yet, such measurement is inadequate in understanding non-Westerners, as it fails to probe the cultural systems underlying this dimension. For example, Chinese people might score high on collectivism, but this may merely indicate a lower degree of individualism in the Western sense. Correspondingly, Fiske (2002) criticizes so-called collectivism as “an abstraction that formalizes our ideological representation of the antithetical other, a cultural vision of the rest of the world characterized in terms of what we imagine we are not” (p. 84).

Nisbett (2004) regards “harmony” as the Chinese counterpart to Greek “agency” and, on this ground, explains why East Asian cultures are more field-dependent/collectivist in orientation, whereas Westerners retain the field-independent/individualist cognitive style of ancient Greece (p. 5). However, Glebkin (2015) challenges Nisbett’s comparison by pointing out that the cognitive style of ancient Greek culture is field-dependent/collectivist when compared to that of the modern West. Therefore, Glebkin suggests a universal multilevel model of a mental structure where the field-dependent/collectivist cognitive style occupies a deeper level than that of the field-independent/individualist, thereby integrating Nisbett’s cultural dichotomy into the different levels of a structure.

The unilateral and oversimplified use of terms from different cultures is an obstacle to the study of self-cultivation. Some ontological premise must have underlain any such investigation, even implicitly, and yet, paradoxically, the implicit nature of these ontologies is, in part, the source of the dilemma of cross-cultural research (Slife and Richardson, 2008). An explicit, and thus examinable, ontology would be more beneficial for research than an implicit one. An ontological model defines interdependent properties and relationships across categories and ideas, and serves multiple functions, including supporting norms, sharing knowledge, and making ontological commitments to address the incommensurability problem (Feyerabend, 1981, p. xi).

Some researchers have adopted specific traditional models of self-cultivation as the ontological bases of related scientific research. A remarkable example of this is Edward Canda applying the Ten Oxherding Pictures of Zen to guide and evaluate social workers’ spiritual development (Canda and Gomi, 2019). The Ten Oxherding Pictures of the 12th-century Chinese Zen master Kuoan Shiyuan describe an ox herder (representing the self) searching for his ox (symbolizing the primordial nature of the self), which remains one of the best models for explicating the awakening process. However, a conceptual model directly stemming from a specific religious tradition is unavoidably tied to the corresponding religious ontology. Therefore, it barely crosses the cultural boundaries to be understood in a consensual way unless transformed into an integrative-philosophical or scientific model with an examinable ontology. Psychological variables hardly explain behaviors in different cultures without the ontological commitment to a cross-cultural ontology. Accordingly, there is an urgent need to develop a preliminary cross-cultural ontological model and ground the research in it, rather than to unilaterally create measuring instruments without a clear understanding of what needs to be measured.

To address this requirement, researchers have suggested methods of adopting universals (e.g., universal cognitive mechanisms) from psychology, biology, anthropology, and linguistics to analyze the underlying structures of cultural systems (Shweder et al., 2006; Genkova, 2015; Bhatia, 2019). However, Archer (1995) asserts that a “fallacy of conflation” in methodology occurs when a psychological theory on cultural phenomena confuses “cultural systems” with “socio-cultural interaction” (p. 58). Therefore, a cultural system cannot be explored *via* empirical research methods such as experiments, questionnaires, and interviews. Instead, the initial steps must include the adoption of humanities research methodologies to analyze the cultural morphostasis/morphogenesis in classics (Archer and Elder-Vass, 2012) and thereby overcome the limitation of positivism that allegedly sustains the objectivity of science but violates the irreducibility of perspectives. Thus, an appropriate cross-cultural ontology can be formed through a contrastive analysis of diverse cultural systems and retroductive argumentation that makes non-linear inferences about the underlying structure of phenomena (Bhaskar, 1975).

### 1.3. An epistemological strategy for developing the required ontological models

To construct the ontological model required to resolve this dilemma, this study adopts Hwang’s (2014) epistemological strategy to develop “culture-inclusive theories” that explain the social behaviors in a given culture (p. 40). This strategy follows the principle of “one mind, many mentalities” (Shweder et al., 2006, p. 871): the deep structures and functions of the human mind are the same across all cultures, while the mentalities develop differently in accordance with the respective cultures. Thus, the strategy adopted is a two-step one. The first step focuses on the “one mind” (universal human mind) and constructs an ontologically universal model as an integrative framework. The second step uses this framework to analyze and integrate specific cultural systems and develop additional culture-specific ontological models. Indigenous psychologists can use these culturally specific ontologies to develop culture-inclusive theories of their own and profoundly probe the “many mentalities.” Both universal and culture-specific ontological models are required for this approach. An explicit example of the culture-specific model constructed using this strategy is the Jun-zi Self-cultivation Model proposed by Xu et al. (2022). They apply Hwang’s (2011) Mandala Model of Self (MMS) as a universal framework to compare, interpret, analyze, and integrate the traditional models of self-cultivation in the Chinese classics *I Ching* (易經) and *Tao Te Ching* (道德經), aiming to transform the cultural system into a psychological theory.

The contradictory perspectives on spiritual hierarchies bring about some methodological considerations essential to constructing the universal model in the first step of Hwang’s epistemological strategy. Friedman et al. (2010) reviewed current ontological models of spiritual development to differentiate “vertical models” (or “stage theories”) that establish development hierarchies from “horizontal” ones that abandon any predetermined spiritual ranking and regard spiritual development as “a horizontal expansion of self-concept” (p. 79). For example, Fowler’s (1981) vertical model of faith development, which assumes a nearly invariant and culturally universal sequence of seven stages, has developed into a robust empirical research tradition. However, as Friedman et al. (2010) point out, this research rests on Western concepts of spirituality and transcendence and may not be universally applicable (p. 88). Wilber’s (2007) integral theory, as a transpersonal perennialist model, provides an overall ontology that integrates the development stages of major traditions into a coherent framework. It is considered the “most impressive example of a vertical model” (Friedman et al., 2010, p. 86). However, based on Ferrer’s (2011) criticism of the pre-established hierarchical rankings of spiritual traditions, states, and orientations, Wilber’s model ontologically entails the “dogmatic privileging of a single tradition as paradigmatic” and thus brings paradigmatic limitations into further theory construction and empirical research (p. 2). By contrast, Ferrer’s (2011) horizontal model reframes human spirituality as emerging from people’s “co-creative participation” in a generative power of reality (p. 2).

Considering these contradictory perspectives, this study draws on Ferrer’s (2011) criticism of Wilber’s perennialist approach as a complementary perspective to self-cultivation modeling. This has two equally important guiding principles. First, the construction of the required universal model should fulfill the first step of Hwang’s strategy by adopting an appropriate form of dialectics, rather than a specific dogma or empirical observation, to “vertically unfold” the layers of self-cultivation sufficient to include all essential and irreducible domains. Root metaphors (such as *taiji* mentioned below) borrowed from various traditions are often used to symbolize a substantive reality of phenomena or the fundamental entity that founds all other entities. However, as the sixth-century Madhyamaka philosopher Bhāvaviveka argued, the asserted foundational entity that is supposedly intrinsically real is, ultimately, neither substantial nor independent and therefore “non-foundational” (*Madhyamakahrdayakārikā*, see Eckel, 2008, p. 73). Apart from being conceptually composed and dialectically identified in a collective whole, nothing withstands as the substance or foundation of other phenomena (Thakchoe, 2017). Therefore, to avoid hypothesizing controversial fundamental entities, the construction of the required model should be dialectical; all ontological domains should be interdependently defined in the whole model and thus be non-foundational. Second, the construction should unfold the ontological hierarchies on an appropriate theoretical model of the self that analyzes the universal ground of human motivations and bridges the subject-object dichotomy, which is the limitation of the epistemology associated with the subject-object ontology inaugurated by Descartes.

### 1.4. Previous psychological models of self-cultivation and their limitations

There are three psychological models of self-cultivation that adopt a specific form of dialectics to unfold Eastern traditions’ self-cultivation stages vertically. The first, the Taiji Model of Self (Wang et al., 2019), divides self-structure into *yin* and *yang—*etymologically, “the shady and sunny side of the mountain” (Stein, 2010, p. 63). *Yin* represents the “small self” that serves the interests of the minority, while *yang* represents the “large self” that serves the majority’s interests. Similarly, in the second model, the Taiji Model of Taoist Self (Wang and Wang, 2020), *yin* represents the “soft self” that reflects “softness, simplicity, non-doing, emptiness, and nature,” while *yang* represents the “hard self” that reflects “hardness, complexity, action, fullness, and artificiality.” In the third model, the Taiji Model of Buddhist Self (Wang and Wang, 2020), *yin* represents the “dusty self,” clinging to the “five root annoyances,” while *yang* represents the untroubled “pure self.” These three models differentiate the self-development process into four or five realms in accordance with the degree of harmony between *yin* and *yang*.

The three models adopt the reductionist paradigm of a dual or dialectical self (e.g., the small and large selves). This approach is oversimplified because it still defines the self in a subject-object dichotomy, similar to that in the Cartesian model of substantial existence of the self. It interprets *yin* and *yang* as dualistic domains, thus failing to exploit two other concepts essential for bridging the subject-object dichotomy: namely, *taiji—*the ultimate from which *yin* and *yang* originate—and *wuji—*best translated as “the limitless” (Ching, 2000, p. 15; Zhang and Ryden, 2002, p. 71). However, *yin*, *yang*, *taiji*, and *wuji* form a set of root metaphors of Chinese metaphysics; therefore, they ontologically imply each other and are mutually manifest. Accordingly, these three models are neither sufficiently comprehensive ontologies of self-cultivation nor applicable as prototypes of the required ontological model.

Theoretical models founded on a subject-object dichotomy cannot encompass the Buddhist notion of “no transcendence” (the term “transcendence” used here being similar to the classical idea of transcendence in Greek-inspired traditions rather than other diverse meanings of transcendence, such as “inward transcendence” used by some Eastern philosophers) or the nondualistic mutual implication of subject and object. Consequently, this study initiates a preliminary development of the ontological model of self-cultivation in line with Hwang’s MMS, which fits the form of Buddhist tetralemmic dialectics that helps incorporate diverse worldviews (delineated in Subsection 2.3). The MMS is inspired by the Borobudur mandala, a massive Buddhist monument in central Java, Indonesia (Hwang, 2011). Viewed from above, the multilevel Borobudur takes the form of a tantric mandala that symbolizes the nature of the mind and the dialectical process of self-cultivation. The central dome of the Borobudur, surrounded by 72 Buddha statues with different *mudras* (symbolic hand gestures), represents the kernel and goal of self-cultivation—great harmony. Founded on a holistic worldview that dismantles the reified subject-object dichotomy, the MMS has laid the groundwork for developing cultural psychology in non-Western countries (Hwang, 2011; Shiah and Hwang, 2018). Based on the MMS, Xu et al. (2022) propose the Jun-zi Self-cultivation Model to outline the process through which an individual (Chinese: 小人; *xiao-ren*) becomes an ideal person (Chinese: 君子; *jun-zi*). The model focuses on presenting the progressive state of *jun-zi* rather than analyzing the structure of the self, but it does not outline the transcendent or immanent domains essential for building an ontology of self-cultivation.

### 1.5. The present work: A dialectically constructed ontological model

The primary ontological model of self-cultivation that deals with all essential and irreducible domains can be developed by dialectically extrapolating Hwang’s MMS into a multilevel model.

**Figure 1:**
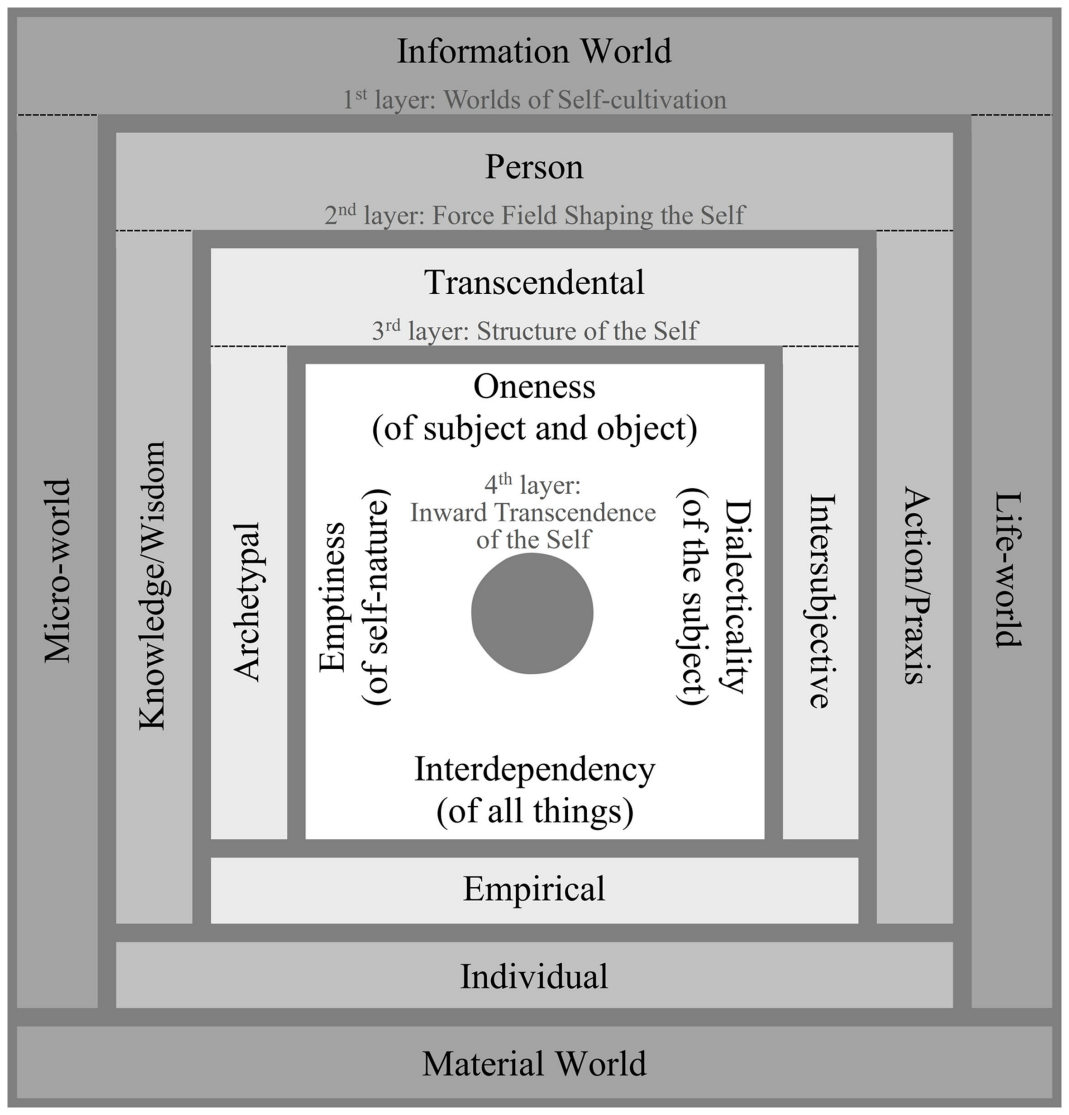
The Dialectical Mandala Model of Self-cultivation.

This study bridges the subject-object dichotomy by using a tetralemmic dialectic, the Four-layered Catuṣkoṭi Framework proposed by the Madhyamaka master Jizang (549–623), to construct a multilevel framework (Figure 1) called the Dialectical Mandala Model of Self-cultivation (DMMS). It proposes a primary model that dialectically unfolds the essential, irreducible, and universal ontological domains of self-cultivation. Responding to the 16 domains (presented in Figure 1) of the DMMS, it recognizes, compares, and synthesizes the representative terms from diverse cultural systems, particularly those of Buddhism, Taoism, and Confucianism.

The DMMS identifies the implications of the 16 self-cultivation-related domains of discourse in diverse cultures. The remaining paper presents it as follows. Section 2 describes the Buddhist tetralemmic dialectics that serve as its framework. Sections 3 –6 define the 16 DMMS domains of the four layers of the model, and section 7 presents the conclusions.

## 2. The dialectic approach to constructing the DMMS

### 2.1. Eastern dialectics as a framework of analysis

As mentioned in the Introduction, a research perspective based on a subject-object dichotomy is inconsistent with most self-cultivation traditions and has hindered the development of an ontological model that integrates knowledge from them. As such, this study employs Eastern dialectics, particularly the catuṣkoṭi (literally “four alternatives,” colloquially “tetralemma”) of subject, object, both subject and object, and neither subject nor object that surpasses the limitations of dualistic and reductionist logic, and the dialecticism of the mutual implication of subject and object, expressed in Chinese by *yin*, *yang*, *taiji*, and *wuji* (the shady, sunny, ultimate, and limitless).

The “naïve dialecticism” (Peng and Nisbett, 1999, p. 744) of acceptance of contradiction is also manifest in the *Bhagavad-Gita*, as noted by Zaehner (1973):

Arjuna, like most Europeans, thinks in either/or categories: he has not yet realized that Krishna’s categories and those of the religion he inherits and further develops are not either/or but both-and. Opposites do not exclude each other but complement each other. (p. 200)

The catuṣkoṭi is a typical Eastern dialectic (Robinson, 1957; Jayatilleke, 1967) that is different from two-valued logic. Nagarjuna provides a typical example:

All is real, or all is unreal, all is both real and unreal, all is neither unreal nor real; this is the graded teaching of the Buddha. (Siderits and Katsura, 2013, p. 200)

Classical two-valued logic insists that a thing cannot simultaneously have opposite attributes; however, Eastern dialectics disagree. For example, in Eastern dialecticism, “existence” and “nonexistence” do not exhaust all possibilities; “both existence and nonexistence” and “neither existence nor nonexistence” coexist. As Matilal (1986) argues, in Buddhism, existence and nonexistence cannot be interpreted by the classical two-valued logic; the syntax of the Buddhist affirming-negative (Skt. *paryudāsa-pratiṣedha*) differs from classical logic (Verhagen, 1994).

The catuṣkoṭi dialectic helps analyze, complement, and integrate worldviews, especially those from the East. This is why, when Buddhism encountered Taoism in ancient China, the catuṣkoṭi could respond to the four core Taoist concepts of *yin*, *yang*, *taiji*, and *wuji*, thus incorporating into its framework a worldview of the mutual implication of subject and object (Chang, 2018). It is also why the construction of the DMMS employs the catuṣkoṭi as the required cross-cultural framework.

### 2.2. Visualization of the catuṣkoṭi

**Figure 2:**
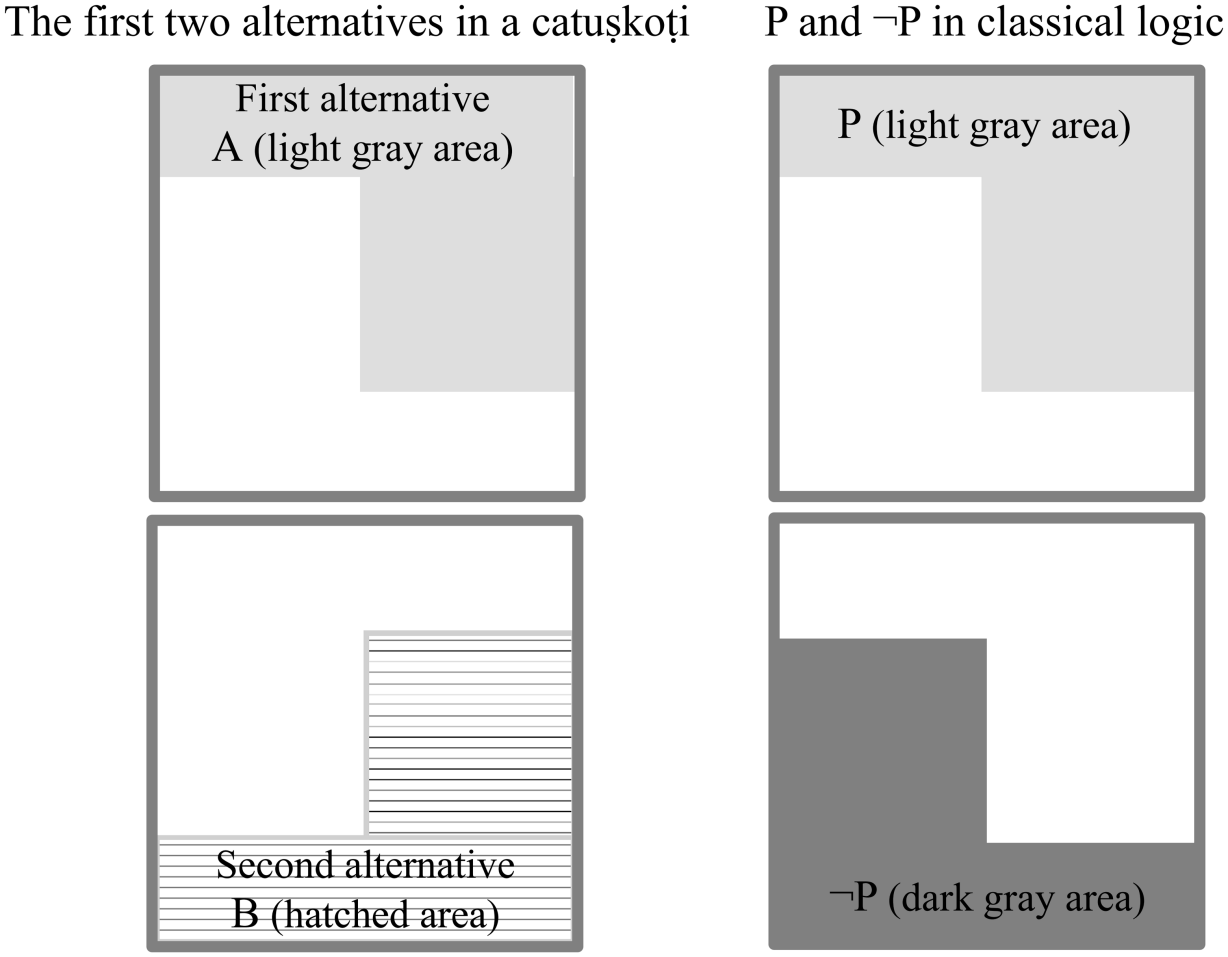
Comparison of the first two alternatives (A, B) in a catuṣkoṭi and a contradictory pair of propositions (P and ¬P) in classical logic.

In classical logic, P and ¬P constitute a pair of propositions that cannot both be true (“law of contradiction”); however, in a catuṣkoṭi, the first alternative (A) and the second alternative (B) might coexist in a superimposition state (Figure 2).

The right side of Figure 2 demonstrates the law of contradiction. The left side shows that the first and second alternatives in a catuṣkoṭi are reversed; thus, they overlap, and both are true.

**Figure 3:**
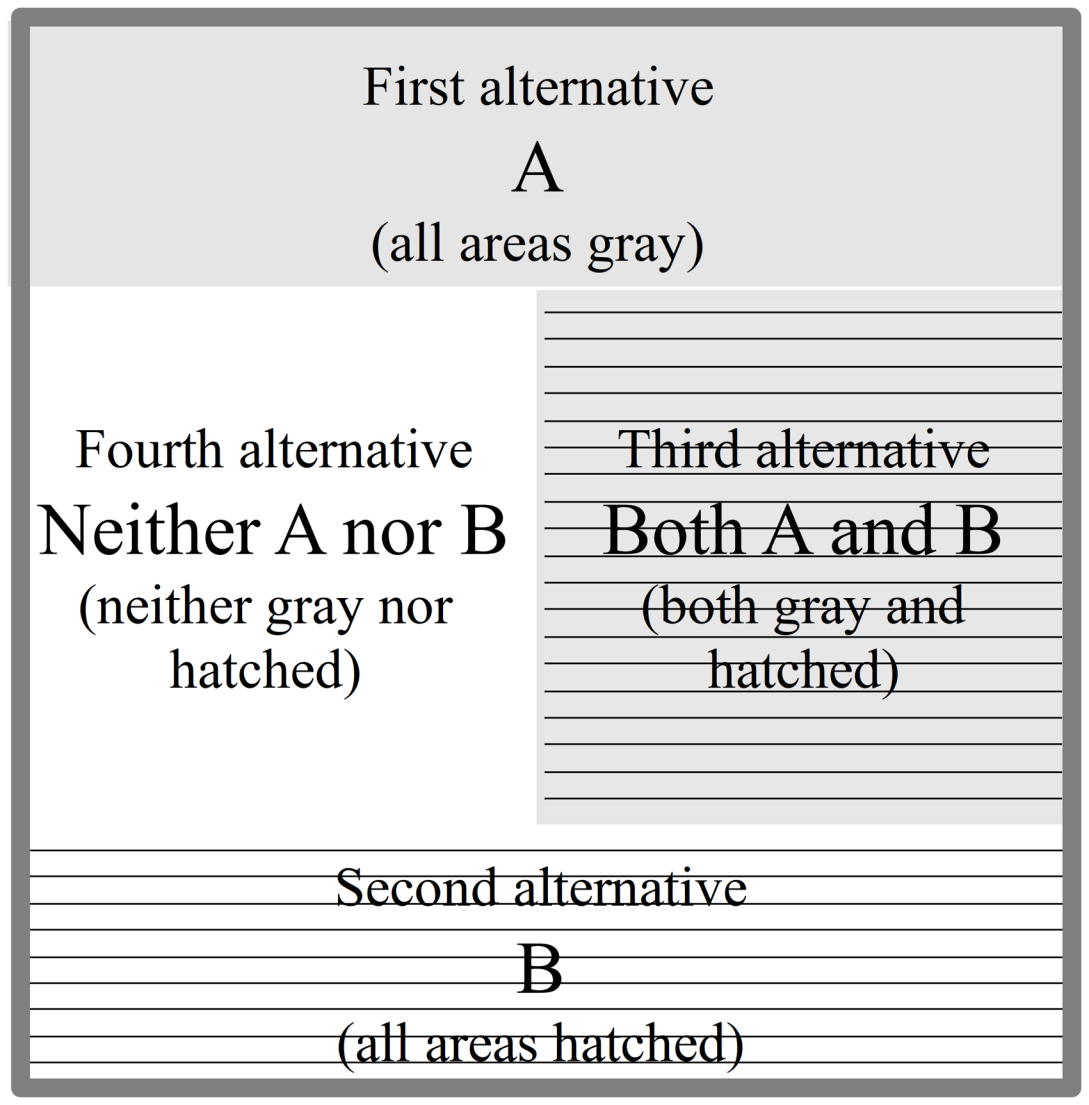
The four alternatives of a catuṣkoṭi.

The catuṣkoṭi comprises four statements expressing four logical possibilities—something is, is not, both is and is not, and neither is nor is not. These statements encompass the main possible objects of a specific discourse or subject matter. In Figure 2, A is a predicate (or a proposition) first proposed as the first alternative (Skt. *koṭi*) in a catuṣkoṭi to represent a specific domain of discourse. Then, B is the second alternative derived from the antithesis of A. C, as the third alternative (“Both A and B,” the overlapping area in Figure 3), represents the compound or integrative function of A and B. By contrast, D (the fourth alternative, “Neither A nor B” in Figure 3) means both A’s and B’s sublation or objectifying awareness.

### 2.3. Analysis and visualization of the Four-layered Catuṣkoṭi Framework

The Four-layered Catuṣkoṭi Framework proposed by Master Jizang in *Commentary on the Vimalakīrti-nirdeśa-sūtra* (T. 38, p. 913a) can serve as a multilevel framework for analyzing the relevant ontological foundations and epistemological orientations of a complex research topic (Chang, 2018). The dialectical principle and construction method of this analytical framework are as follows. A catuṣkoṭi comprises four alternatives analyzing four representative domains of a specific discourse so that the four alternatives represent all possible domains of this discourse. As the analysis deepens, it extends to a parallel discourse that transcends yet integrates all the previous domains, thus forming a higher-layer catuṣkoṭi in a multi-layered catuṣkoṭi framework. For analyzing a complex topic, four layers of catuṣkoṭi are built to explore different relevant discourses, such as the phenomenal, methodological, epistemological, and ontological discourses (Chang, 2018).

**Figure 4:**
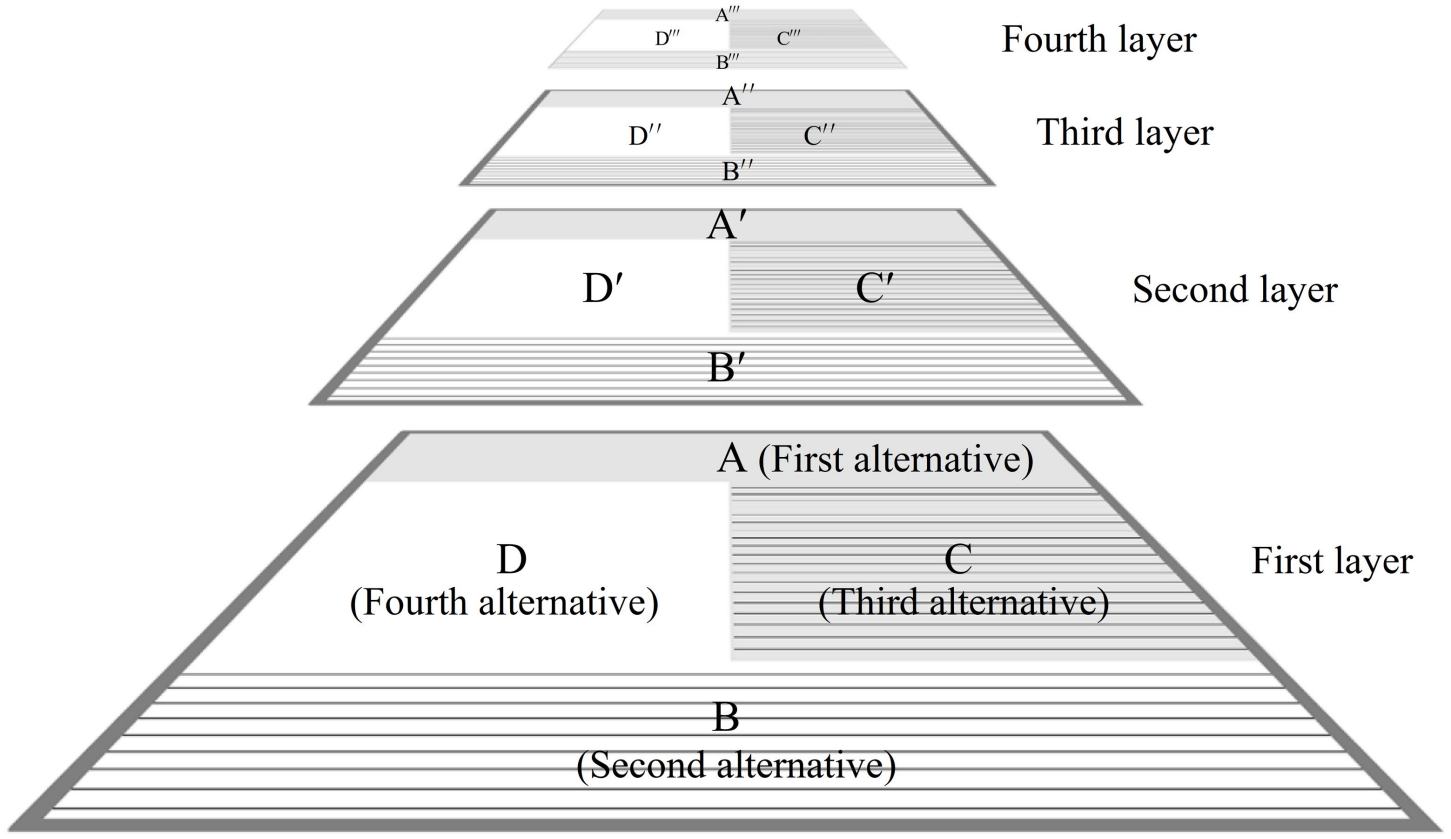
The three-dimensional structure of the Four-layered Catuṣkoṭi Framework.

In the case of the DMMS, the first-layer catuṣkoṭi’s four domains (A, B, C, and D in Figure 4) represent all the worlds of self-cultivation. In the Taoist worldview, they would correspond to the root metaphors of *yin*, *yang*, *taiji*, and *wuji*: the information world, the material world, the life-world (from which *yin* and *yang* originate and to which they return), and the mind world (without the limitation of *yin* and *yang*), respectively. The four worlds can be integrated as a person’s integral world consciousness. Therefore, the domain of the person, the first alternative of the second-layer catuṣkoṭi (A′ in Figure 4), is the integrator of the whole first-layer catuṣkoṭi. In other words, after establishing A, B, C, and D in Figure 4, it obtains A′, which integrates all four alternatives of the previous layer. Subsequently, B′, C′, and D′ of the second-layer catuṣkoṭi are dialectically developed from A′. In this manner, the second-layer catuṣkoṭi is dialectically derived from the first-layer catuṣkoṭi. The same method forms the third and fourth layers. Figure 4 shows the complete Four-layered Catuṣkoṭi Framework.

**Figure 5:**
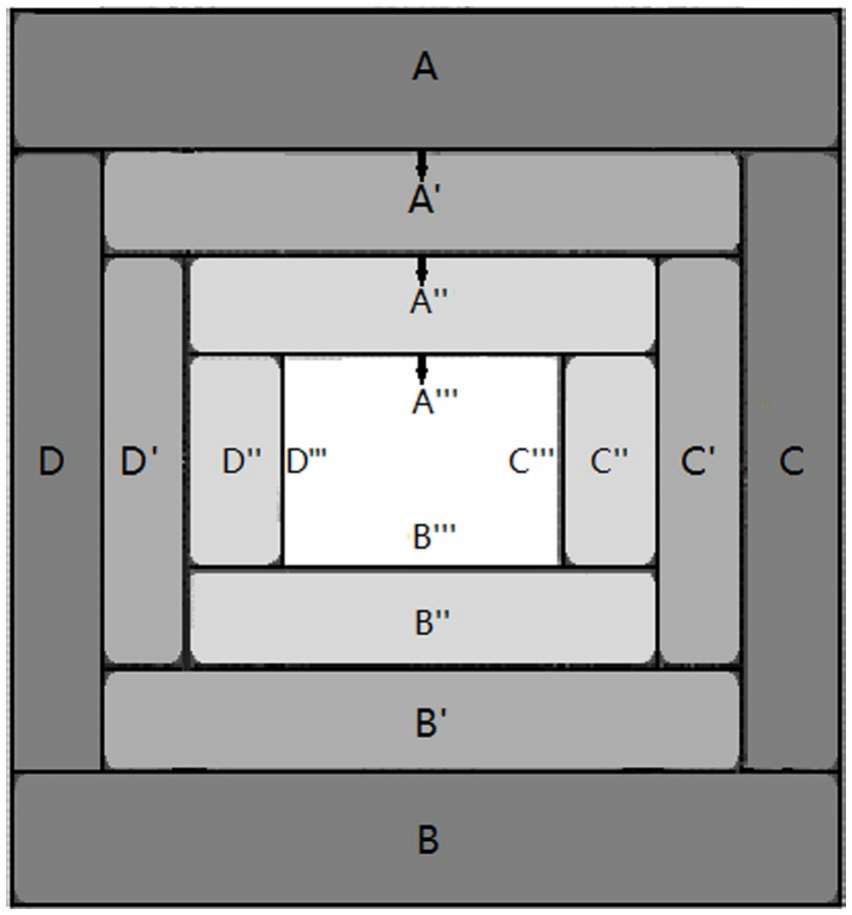
Two-dimensional visualization of the Four-layered Catuṣkoṭi Framework.

To tabulate the 16 domains of the four layers, the DMMS depicts the top view of Figure 4 with a slight simplification and modification, taking the form of the Buddhist womb mandala (Garbha Maṇḍala), as shown in Figure 5. The first and second alternatives of the first-layer catuṣkoṭi (A and B in Figure 3) are reduced to the upper and lower rectangles (A and B in Figure 5). The third alternative (“Both A and B” in Figure 3) is represented by the rectangle on the right (C in Figure 5) because the second layer overlaps with the center of the first layer. Likewise, the fourth alternative (“Neither A nor B” in Figure 3) is reduced to the left rectangle (D in Figure 5). The same method is used for the second and third layers.

## 3. Defining the first DMMS layer: The worlds of self-cultivation

Chinese thinkers tend to discuss the effort of self-cultivation in the context of a body–mind continuum (Huang, 2017). To transcend the subject-object dichotomy, Velmans (2017) proposes reflexive monism, a kind of mind–body monism, and links the theory to Advaita nondualism. He suggests that the body and mind are two aspects of something that is in itself neither mental nor physical, similar to Leibniz’s monad. Such mind–body monism has developed in several directions, including different divisions of emergentism (e.g., Searle, 2002; Kauffman, 2008) and panpsychism (e.g., Skrbina, 2005; Seager, 2006). Panpsychism postulates a protomental nature that forms the substrate of the universe, matter, and consciousness (Griffin, 1997), thus echoing the Eastern concept of self-nature (Skt. *svabhāva*), which refers to the intrinsic nature or essence of beings (Hwang, 2019; Wang and Wang, 2020). As a variant of proto-panpsychism, Pereira’s (2013) triple-aspect monism (TAM) further differentiates between the unconscious mental aspect (i.e., the “informational aspect”) and the conscious mental aspect (p. 328).

**Figure 6:**
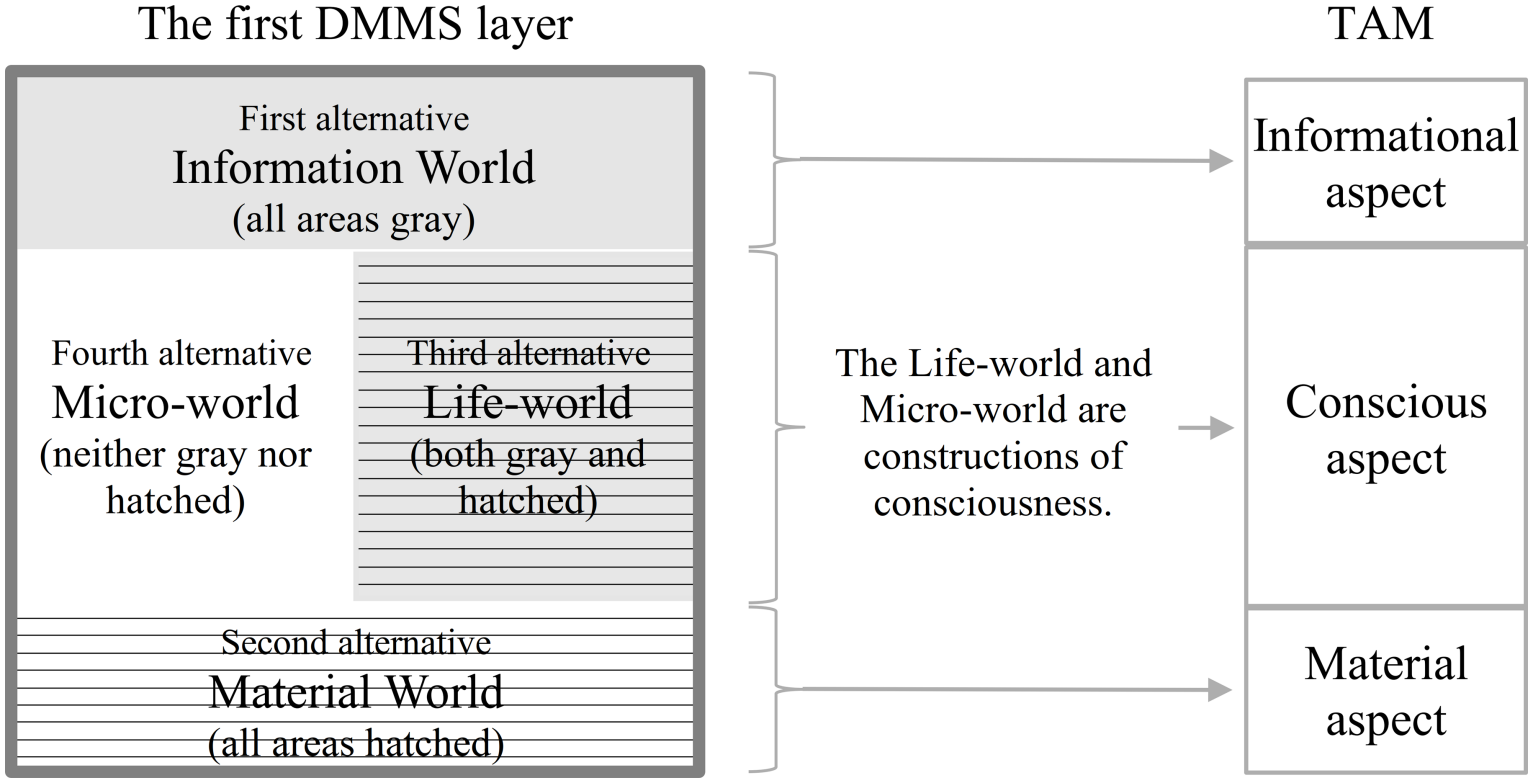
The first Dialectical Mandala Model of Self-cultivation (DMMS) layer mapped onto the triple-aspect monism (TAM) framework.

Based on a *yin-yang* model, Chinese cosmology forms an understanding of the *yin* world (*yinjian*; the nether world) in contrast to the *yang* world (*yangjian*; this world). First, the *yin* world is an information world that registers the life experiences of all beings since the beginning of time (Lee and Chang, 2001). The information world is set as the first alternative of the first DMMS catuṣkoṭi. Second, the material world is the second alternative, the antithesis of the information world. Third, *yin* and *yang* together produce everything that constitutes our life-world (the third alternative). Fourth, to face, comprehend, and explain the given world, one constructs the world of comprehension, the micro-world (the fourth alternative). This study maps the first DMMS layer onto the TAM framework (Pereira, 2013), as shown in Figure 6.

The first and second domains of the first DMMS layer map onto TAM’s informational and material aspects, respectively. Although the third and fourth domains map onto TAM’s conscious aspect, the first DMMS layer does not merely identify consciousness; instead, it determines whether the construction of consciousness is collectively inherited (the third domain) or is original (the fourth domain). It identifies the two constructs of consciousness: (1) the life-world collectively constructed and inherited by people as “beings-in-the-world” (Dreyfus, 1990, p. 40) and (2) the micro-world constructed from one’s observation, analysis, and comprehension of the world.

**Figure 7:**
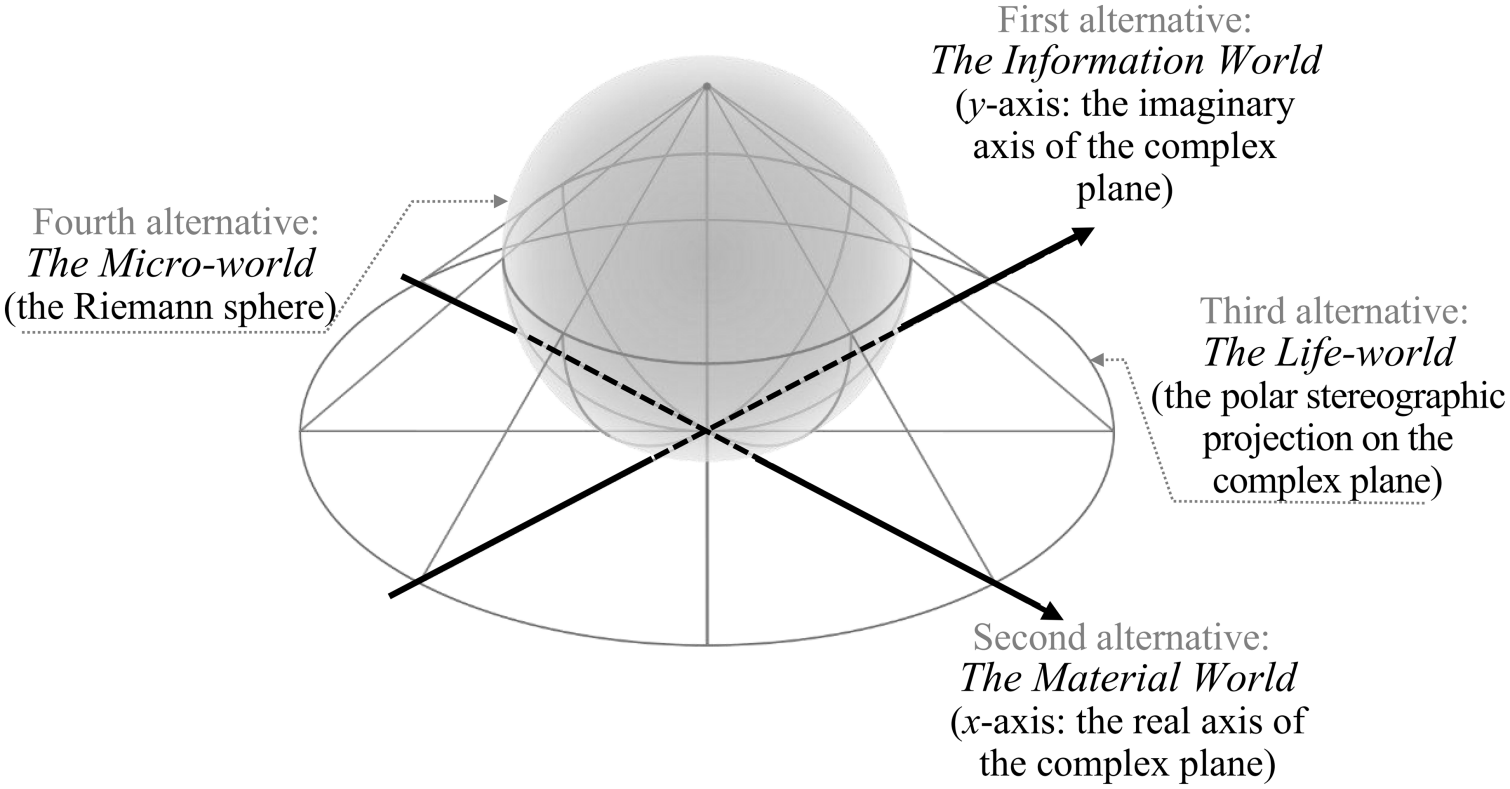
The catuṣkoṭi structure of the quadruple worlds.

The catuṣkoṭi structure of the four worlds can be figuratively conceived as a stereographic correspondence between the Riemann sphere and the complex plane (also called the Argand plane), as shown in Figure 7. The Riemann sphere, named after Bernhard Riemann (1826–1866), is a mathematical model mapping the extended complex plane. The *Avataṃsaka* master Fa-tsang (643–712) illustrated the doctrine of the manifestation of the Buddha-mind (Skt. *Tathāgatôtpatti-saṃbhava*) using the simile of Indra’s net of pearls. All pearls are reflected within each pearl of Indra’s net and constitute a monad-universe in which all beings are connected by infinite threads of relationality in nonspace and nontime (Liu, 1982). Fa-tsang’s distinction between the Buddha-mind and its manifestation roughly corresponds to Leibniz’s distinction between the monad-universe and the perceived universe (Liu, 1982). Indra’s net of pearls and the Buddha’s third eye are all archetypes of the Buddha-mind, analogically modeled as a monadic sphere to represent the *Avataṃsaka* micro-world. All beings interdependently exist and are essentially equal; they are equally represented as points on a sphere. Each point on the sphere has a tangent plane passing through it, which serves as the base plane of the model, containing the real and imaginary axes that represent the material and information worlds (Lee, 2019), respectively. The stereographic projection onto the plane represents the life-world.

The definitions of the four first-layer DMMS domains are presented in the following subsection.

### 3.1. The information world

An information pattern is transmittable from one material system to another. In TAM (Pereira, 2013), consciousness “goes away” at the moment of death; however, some of the elementary mental forms continue and are “re-actualized by other individuals” (p. 323). Pereira (2013) calls these transmittable potential mental forms “information” (p. 313). The definitions and explanations of TAM’s informational aspect are consistent with modern scientific research and provide a framework to study karma seeds, *saṃsāra*, and causal agents (e.g., deities, spirits, fate, and astrological influences).

Corresponding to the informational aspect in TAM, the information world defined in the DMMS is unconscious. However, mystics and enlightened masters have been meditating since ancient times to gain insight into the unconscious information world. According to Yogācāra Buddhism, all experiences produce karmic seeds (Skt. *bījas*) as impressions, which are stored in *ālaya-vijñāna* (repository consciousness).

### 3.2. The material world

Eliade (1959) notes that for a person on a spiritual path, “nature is never only ‘natural’; it is always fraught with a religious value” (p. 116). Everything in the material world is inherently unstable in its transience but offers an opportunity to realize life as an ego-transcending practice. In the past few decades, some religions (e.g., Tibetan Buddhism) have begun a dialog aimed at integrating natural science and religion. For example, electroencephalographic studies of the effects of different types of meditation help advance our understanding of self-cultivation traditions. The neurobiological approach to self-cultivation uses emerging technologies (e.g., functional magnetic resonance imaging) to measure the physical expressions of spiritual variables, despite criticisms of reductionism (Velmans, 2017).

### 3.3. The life-world

Husserl’s (1970) life-world is a phenomenological concept that refers to “wakeful world-consciousness” and the self-evident world experienced by people (p. 108). Various religions are grounded in an original unity of life-world consciousness that precedes the subject-object dichotomy. Life-world construction comprises unconscious information processing (the first domain) and material mechanisms (the second domain). The information-material duality of the life-world is analogous to the wave-particle duality of quantum consciousness (Di Biase, 2013; Hameroff and Penrose, 2014). Thus, the life-world is identified as the third alternative of the first DMMS catuṣkoṭi.

Life-world construction is based on historical, cultural, and social consciousness. Therefore, it is neither separable from nor reducible to the information world or material world. When studying self-cultivation experiences in religious organizations or societies, we should analyze the life-world domain, such as a religious organization’s type of authority (e.g., traditional, charismatic, legal-rational).

### 3.4. The micro-world

The term “micro-world,” introduced by Wallner (1994), originally refers to a functioning scientific construct. However, in the DMMS, its meaning is extended to represent all the mentally constructed realities that everyone consciously forms. The sentiment that “things are not whole,” as illustrated in the first of the Ten Oxherding Pictures (the Searching for the Ox), drives people to search for the “lost ox,” thereby finding the “ox’s trace” (Trungpa, 2001, p. 74). The search of the world for the tracks as a substitute for the actual ox represents the comprehension and theorization of one’s experience of life—the construction of the micro-world. Based on the life-world and guided by varying themes for different needs, humans have created various micro-worlds of science, ethics, esthetics, and religion (Hwang, 2006, p. 85); although the absolute reality seems beyond human knowledge, people have constructed various micro-worlds to approximate it. One’s beliefs and worldview belong to this domain, and therefore, the result of self-cultivation is associated with one’s micro-world consciousness and should be systematically considered in this domain. For example, to understand the conduct of specific ascetics, the researcher should first probe their micro-worlds of belief.

## 4. Defining the second DMMS layer: The force field shaping the self

**Figure 8:**
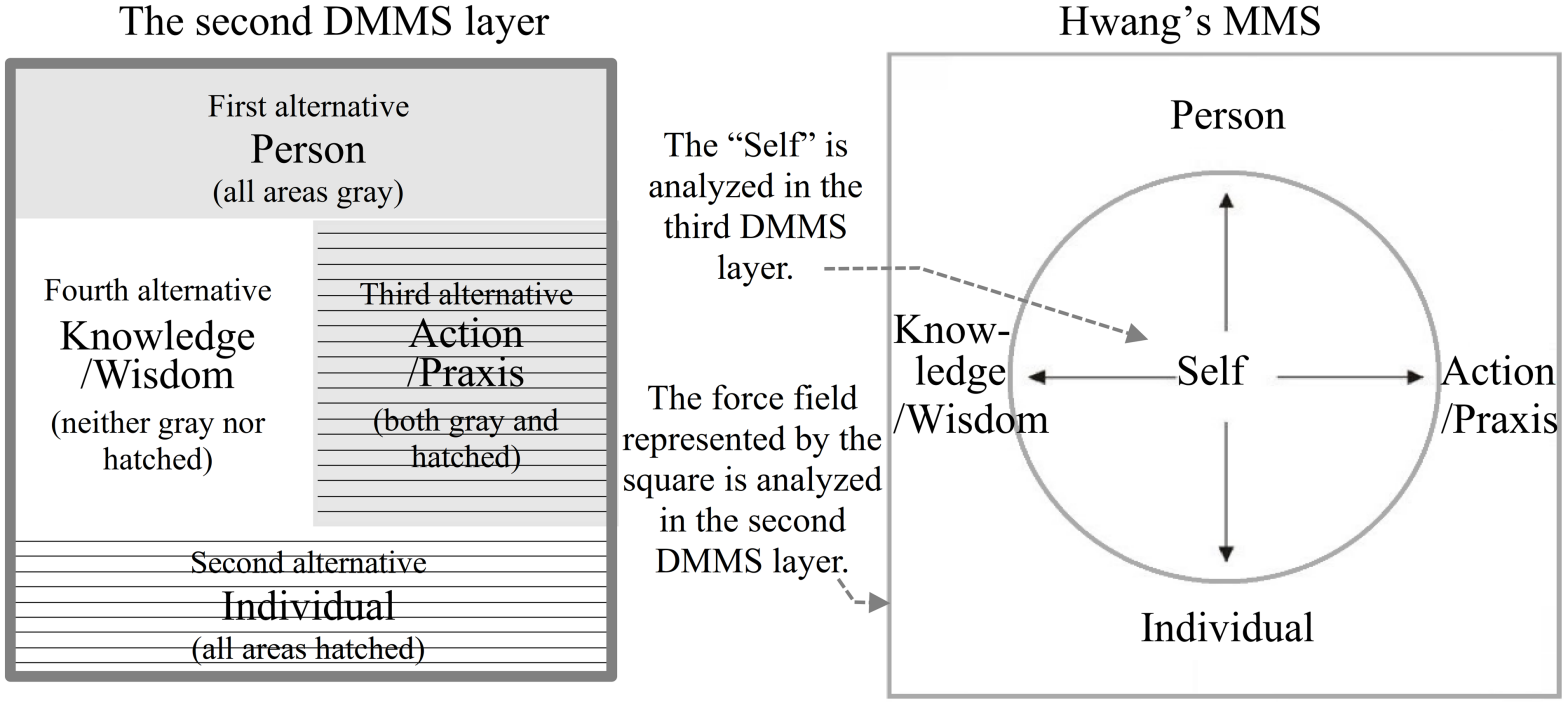
The second Dialectical Mandala Model of Self-cultivation (DMMS) layer and Hwang’s (2011) Mandala Model of Self (MMS).

The second stage of Zen practice depicted in the Ten Oxherding Pictures is Seeing the Traces, which refers to seeing the “conflicts created by the ego” (Trungpa, 2001, p. 76). Correspondingly, the second layer of the DMMS presents the dialectical force field created by and, in turn, shaping the self, aligning with Hwang’s MMS, as shown in Figure 8.

### 4.1. Person

Hwang (2011) defines “person” as “an agent-in-society who takes a certain standpoint in the social order and plans a series of actions to achieve a particular goal” (p. 65). “Person” is a sociological or cultural concept, and its meaning in a specific culture reveals that culture’s values and worldview.

Unlike the Christian view of individual equality, Eastern cultures tend to regard individuals as unequal according to their place in a spiritual hierarchy and the level of self-cultivation they have achieved (Dumont, 1980; Friedman et al., 2010). The Confucian classic *Great Learning* (Chinese: 大學; *Da Xue*) states that “from king to ordinary people, everyone must take self-cultivation as the basis of person-making” (Chinese: 自天子以至於庶人壹是皆以修身為本). To understand this concept of person-making, we may refer to Mencius (372–289 BCE), who believed that “[a] person has these four dimensions (propriety, righteousness, integrity, and a sense of shame) just as having four limbs” (Chinese: 人之有是四端也猶其有四體也) (*Mencius*-*Gong Sun Chou* I: 6). In this sense, a person is a unique individual with a name, history, duties, social roles, hierarchical identity, spiritual development, and worldview. Person-making is essentially about self-cultivation, and a person’s being-in-the-world integrates all four worlds of the first DMMS layer. Thus, “person” is the first domain of the second DMMS layer.

### 4.2. Individual

In the sacred-secular duality of most cultural traditions, the biologically defined “individual” contrasts with the culturally defined person. Thus, the domain of the individual is the second alternative of the second DMMS catuṣkoṭi. An individual, as a biological entity, is driven by various desires (Shiah and Hwang, 2018). Therefore, the egoistic and altruistic variants of hedonistic ethics (including those teaching a pursuit of spiritual bliss or maximizing the pleasure of most people) are rooted in this domain. Contrary to a person’s uniqueness, all individuals are equivalent and reduced to anonymous statistical entities in an empirical study.

### 4.3. Action/praxis

Heidegger calls the activity of existing “being-in-the-world” (Dreyfus, 1990, p. 40). “Being” can be translated as “entity”; however, the word also emphasizes that human existence is a verb rather than a noun. The hyphenation emphasizes the primordial praxis of our existence in the world. People’s actions are rooted in biological (the domain of the individual) and cultural (the domain of the person) drives; the praxis as being-in-the-world integrates the individual with the world that the person stands for. Thus, the action/praxis is the third domain, the integrative function between the individual and personal domains. Finally, acts undertaken for self-cultivation, such as fasting, meditation, prayer, worship, and pilgrimage, can unravel and unfold their systematic meaning from this DMMS domain.

### 4.4. Knowledge/wisdom

Knowledge and beliefs support many human actions undertaken to achieve self-cultivation; the wisdom for action is contained in one’s stock of knowledge (Hwang, 2011). According to Piaget’s (1997) genetic epistemology, knowledge is neither *a priori* truth, as suggested by idealists, nor determined by sensory experience, as argued by empiricists (p. 19). Thus, knowledge/wisdom belongs to neither the domain of the person (the first alternative of the second DMMS catuṣkoṭi) nor the individual domain (the second alternative). As the fourth domain of the second DMMS layer, the knowledge/wisdom domain corresponds with the domain of action/praxis. Through action/praxis, people acquire and modify knowledge/wisdom, and through knowledge/wisdom, they adopt action/praxis.

## 5. Defining the third DMMS layer: Structure of the self

The notion of self plays a significant role in self-cultivation traditions. Metzinger’s (2006) nihilist view suggests that the self, generated from the brain’s message processing system, is phenomenal, has no substance, and does not exist independently of the brain. Vasubandhu explained continuity from one lifetime to the next using the simile of one flame of a fire giving rise to another (*Ātmavādapratiṣedha* 4.3.5–6), thus emphasizing non-self, non-essentialism, and impermanence. However, as Thurman (2005, p. 200) has pointed out, although Buddha proclaimed that an “absolute self” (an independent referent of “I”) does not exist, he taught the co-dependent structure of a “relative self.”

The Vedānta philosophers conceived the individual self (*jiva*) as a “reflection” or a “limited appearance” of *ātman* (the transcendental and highest self; Deutsch, 1980, p. 54). The concept of *ātman* serves as the first principle in all major schools of Hinduism. Differing from the *ātman* concept, the *anātman* (non-self) concept found in some Yogācāra, Yogācāra-Madhyamaka, and Tathāgatagarbha texts reveals that the core nature of consciousness is non-dual reflexive awareness (MacKenzie, 2012). Thus, Mahāyāna Buddhism grasps consciousness as an ever-present self-luminous awareness without making an ontological commitment to *ātman*. However, both traditions identify two aspects of consciousness, the empirical and the transcendental, and link the inherent reflexivity of consciousness to its transcendental aspect (MacKenzie, 2012).

The dual-aspect analysis is analogous to Immanuel Kant’s view of subjectivity, which distinguishes the transcendental subject from the empirical subject. For Kant, the transcendental self is inferred from and serves to unify itself with the existence of the empirical self (Kant, 1787 [1929]). One cannot be conscious of the transcendental self, which is only revealed through Husserl’s (1960, p. 37) transcendental-phenomenological *epoché* (suspending assumptions and beliefs). By contrast, the empirical self is what James (1890, pp. 291–298) calls “the self as known” (an objective “me”).

The DMMS establishes the dialectical existence of the “relative self” (Thurman, 2005, p. 200) *via* the four alternative domains of the third DMMS catuṣkoṭi. The third DMMS layer is an epistemological analysis that differentiates the transcendental domain (the first alternative of the third DMMS catuṣkoṭi; subject not objectified) from the empirical domain (the second alternative; objectified subject) of consciousness to reflect a subject and object’s structure of mutual implication. In the experience of consciousness, the empirical self’s role is that of the subject; the transcendental self, functioning within the empirical self, integrates the force field shaping the self (the second DMMS layer) and associates the fragments of the subjective experiences of different time-spaces and roles, thus supporting united self-consciousness. In the intersubjective domain (the third alternative), the transcendental and empirical selves are integrated into real-life consciousness. As the fourth alternative of the catuṣkoṭi, the archetypal domain reflects the principle of how consciousness emerges from the transcendental and empirical selves. The archetypal self refers to an archetype of order and wholeness that organizes and directs all other elements of our psyche (Jung, 1986), identical to neither the empirical nor the transcendental self. These four domains are explained next.

### 5.1. Transcendental

Since ancient times, people have imagined and pursued a pristine absolute subject that functions as the transcendental unifier of subjective experience. Concepts of the higher self, such as the “unconditioned self,” “whole self,” and “soul” (Holden, 2007, pp. 13–16), are roughly analogous to Kant’s concept of the transcendental self. In fact, notions of the transcendental self vary across traditions. For instance, opposite to fatalism, the innate Buddha-nature (or the Buddha within), proposed by the Buddhist Tathāgatagarbha school as the origin of reflexive awareness and the transcendental handler of karma seeds, exists on the ontological ground of emptiness (Skt. *śūnyatā*). Still, in ontological arguments of Abrahamic religions, the soul is derived from the existence of God, symbolizing the transcendental self whose essence is God. Thus, mystics could represent the transcendental self as a *Deus absconditus*, a hidden God, pointing out the unknowability of the essence of God. Furthermore, Taoists and Confucians meditate on the immortal within, *shen* (Chinese: 神; literally meaning “spirit,” “soul,” and “deity”), another self that is not shaped by embodied experience and thus belongs to the transcendental domain. Whether called Dharmaraja or Jehovah, an impartial God maintaining the law of karma or the existence and equilibrium of the life-world can symbolize the transcendental self.

The third stage of Zen practice depicted in the Ten Oxherding Pictures is Glimpsing, a self-reflexive glimpse into the non-discriminating and non-conceptual transcendental self. The glimpse makes one ascend the first of the 10 Faith Stages described in the *Jeweled Necklace Sutra* (Chinese: 瓔珞經; T. 24, pp. 1017–1023). The transcendental self as “the universe of possible forms of subjective process” (Husserl, 1960, p. 73) is transpersonal (Assagioli, 1973).

### 5.2. Empirical

As the self’s transcendental domain rises and integrates the force field shaping the self (the second layer of the DMMS), the opposite—the objectified empirical domain of the self—is shaped by the force field. In this domain, self-schema, body awareness, dream ego, fantasy self, and an entire collection of dispositions are all knowable *via* introspection and observation. Accordingly, specific self-cultivation systems, such as the silent-illumination meditation (Chinese: 默照禪; *Mozhao Chan*) of Master Hongzhi (1091–1157), utilize meditation on the impermanence of the empirical self as a way to achieve liberation. The sixth of the Ten Oxherding Pictures, the Riding Home, depicts such meditation (Yamada, 2004).

### 5.3. Intersubjective

According to Husserl (1970), one’s experience of objects in the world is necessarily “intersubjective,” which means it is accessible or can be established for two or more subjects (p. 168). Human consciousness comprises intersubjective integration of the empirical self’s various modes (e.g., a person’s dream egos in different dreams) and of self-awareness and other-awareness. The idea of intersubjectivity describes such constitutional interdependence of human consciousnesses. Based on intersubjectivity, the subjective experiences become integrated, and self-consciousness emerges (Stern, 2002). Intersubjectivity is an integral aspect of the self, and intersubjective consciousness differs from Descartes’ notion of consciousness as an independent walled-off sphere wherein resides a pristine self (Stern, 2002).

The “social self” (James, 1890, p. 293) that we present and experience as “I” in society and the “transpersonal identities and subjectivities” (Berlant, 1998, p. 283)—for example, the mystical Tibetan tantric practice of awakening to one’s identity as a compassionate bodhisattva—belong to the intersubjective domain of the self. In Mahāyāna Buddhism, the bodhisattva’s Nirmānakāya (literally “transformation body”; one of the three bodies of the Buddha) is manifested in response to the deep longing of sentient beings. The fifth of the Ten Oxherding Pictures, the Taming, metaphorically depicts such intersubjective responses. In parallel, *Tao Te Ching*, a fundamental text for Taoism, notes that a sage has no invariable mind of his own but makes people’s minds his own (Ch. 49). In specific Taoist rituals, priests perform as spirit mediums for the deceased or deities and comfort believers.

### 5.4. Archetypal

Jung (1986) describes archetypes as “identical psychic structures common to all” (p. 65). Archetypes have universal forms in the collective unconscious and become the contents of conscious experience in response to specific situations. For instance, during the Taoist Qianwang (“guiding the deceased”) ritual, Taoist priests “perceive” clients’ relatives who have passed away, converse with the clients as the deceased, and reveal private matters, intentionally or otherwise. Their conversations often outline the *yin* world and its king, Yanluo, resembling the typical descriptions of the nether world and ruler of death in various myths. Archetypes such as Yanluo become factors of psychological empowerment and trigger human potential to become moral, compassionate, and wise. Sometimes, an archetype manifests as a deity to the believer, with specific images and ideas that construct the individual’s worldview and define good and evil. However, specific transcendental ideas may lead people into contradictions or antinomies. Therefore, a central concern of many religions is to justify their archetypal ideas.

A great variety of archetypes are described in diverse religions as sacred entities, including various astrological formations, mythological characters, and deities. Thus, psychologists explore archetypes through myths, rituals, art, and people’s spiritual experiences. Jung (1986) listed examples of archetypes: mother, father, anima, animus, persona, shadow, and the core archetype—the archetypal self. The fourth of the Ten Oxherding Pictures, the Catching, depicts the ox herder regaining the lost ox and the temporary equilibrium in the archetypal self. However, the DMMS only dialectically identifies the archetypal self in its third layer (the structure of the self), which still falls short of the ultimate reality. The ultimate reality is symbolized by the top and innermost layer, the inward transcendence of the self.

## 6. Defining the fourth DMMS layer: Inward transcendence of the self

Fa-tsang defined the “Round Teaching” (complete teaching) in his *Treatise on the Five Teachings*:

As to the Round Teaching, which is all about the undistorted and holistic nature of everything and the self, the infinite dependent origination, and the unobstructed interpenetration: The one is the many, the many are the one, the subject integrates with the object consummately. (T. 45, p. 485b, ll. 7–9)

The DMMS draws on the Buddhist argument of self-nature being “one, many, both one and many, neither one nor many” (Tillemans, 1984), incorporating round teaching into its fourth layer. It defines the alternatives of the fourth DMMS catuṣkoṭi as follows: the oneness of subject and object (immanent integration of the dialectical structure of the self) as the first alternative of the catuṣkoṭi (the one), the interdependency of all things (transcendent integration between self and other) as the second (the many), the dialecticality of the subject as the third (both the one and the many), and the emptiness of self-nature as the fourth (neither the one nor the many). Accordingly, the DMMS reveals the kernel of self-cultivation on the top layer, termed “inward transcendence of the self ,” as a means to compare, identify, analyze, and integrate the diverse spiritual advancement in various cultural contexts.

### 6.1. Oneness (of subject and object)

According to the subject-object dichotomy worldview, the mind cannot perceive things-in-themselves (*noumena*). However, some scientists have begun to perceive the mind as reflecting the same quantum phenomena that make the physical universe possible (Bohm, 1951; Hameroff and Penrose, 2014).

The holistic immanence, the oneness of subject and object, is an ultimate concern in most self-cultivation traditions, particularly Confucianism, Taoism, and Buddhism. According to the Confucian classic *Doctrine of the Mean* (Chinese: 中庸; *Zhong Yong*), “complete sincerity” (至誠) assists in “Heaven and Earth’s transformation and sustenance” (天地之化育). In other words, a sincere human can merge with the infinite Heaven and Earth.

Similar doctrines describe the oneness of subject and object. The *Avataṃsaka*’s Mind-Only Poem points out the oneness of the mind, the Buddha, and all living beings and reflects the influence of Yogācāra’s mind-only (Skt. *citta-mātra*) philosophy that disaffirms the existence of external objects.

The Buddha is also like the mind, and living beings are like the Buddha. The mind, the buddhas and the living beings—there is no difference between these three. (Mind-Only Poem, T. 9, p. 465c, ll. 28–29)

If one understands that the activity of the mind creates the worlds everywhere, he will see the Buddha, and understand the real nature of the Buddha. (T. 9, p. 466a, ll. 1–2)

In essence, the mystical understanding of the holistic oneness (or mind-only) described above is an advanced stage of self-cultivation in which one’s sense of a separate self is abandoned. Since ancient times, “to see one’s own originally enlightened mind” (Chinese: 明心) and “to see the self-nature” (Chinese: 見性) have been key to becoming a saint. The seventh of the Ten Oxherding Pictures, the Transcending Other, depicts the ox herder’s awareness of oneness.

### 6.2. Interdependency (of all things)

Antithetical to the one (oneness of subject and object) is the many (interdependency of all things). Through transcendence, one sees the connectedness underlying the diverse elements of nature, that is, the interdependency of all things.

The interdependency of all things has two dimensions. The first one argues that the existence of everything depends on one God, whereas the second dimension asserts that everything is interdependent, as in the Buddhist concept of “dependent origination” (Skt. *pratītya-samutpāda*). In Mahāyāna Buddhism, interdependency does not harbor a subject-object dichotomy worldview. The image of “all Buddha fields contained in one atom” (e.g., T. 10, p. 906c, ll. 25–26), often repeated in the *Avataṃsaka*, led ancient masters to formulate a sophisticated philosophy of interdependency (McMahan, 2002). Thus, the final stage of enlightenment depicted in the Ten Oxherding Pictures is an awakened state of being-in-the-world, akin to “the moon reflecting in a hundred bowls of water” (Trungpa, 2001, p. 92), capable of seeing the Buddha-nature in all beings (Yamada, 2004). Mahāyāna philosophy posits that a lucid insight into self-nature illuminates the interdependency of all beings, which extinguishes the self and conquers death anxiety, thus leading to a state of “nonself-plus-compassion” (Shiah, 2016). Such a keen awareness of interdependency might have been echoed by the quantum mechanical discovery of the interaction between the observer and the observed. However, the observer’s role in the collapse of the wave function into discrete particles might be construed as actually affirming the centrality of the self.

### 6.3. Dialecticality (of the subject)

Hegel’s (2010) dialectic, which serves as “the principle of all natural and spiritual life” (p. 35), asserts a conceptual progression toward a teleological end in absolute spirit; thus, the self-estrangement process (self-objectification and alienation) may be part of self-creativity and self-discovery (Vaughan, 2015). There is a similitude between the Hegelian immanent teleology of absolute spirit and the Mahāyāna teleology of the dialectical manifestation of Buddha-nature in that the means (the dialectical process) to the end (absolute spirit or Buddha-nature) is inherent in the end; that is, the unpredictable end is the subject achieving itself in the means (Kim, 1996, p. 66).

As some individuals approach the top of Maslow’s (1971) hierarchical model of needs with a strong motive toward alleged self-transcendence, they identify with something more authentic than the purely individual self and engage in a form of self-cultivation, perhaps through transpersonal or mystical experiences (Koltko-Rivera, 2006). Wilber (2007) holds that people’s self-consciousness and reality are evolving in an immense “natural hierarchy,” an “order of increasing wholeness” (p. 25). Self-cultivation tends to be a dialectical process of moving from one stage of identification with the lower self to a transcendental identification with the transpersonal self. Defined as an integration of the one (the first alternative of the fourth DMMS catuṣkoṭi) and many (the second alternative), the dialecticality of the subject (the third alternative) manifests the integral subjectivity in a dialectical process, such as the process of awakening from seeking the manifested (the many) to realizing the concealed Buddha-nature (the one).

This domain of self-cultivation circumvents monotheism and pantheism, grounding two forms of spiritual practice. In the first, a human being achieves the spirit of transcendence with complete surrender to a transcendent being. In the second, the empirical self inwardly integrates with the transcendental self, as in the “metamorphic transformation” of a person into an immortal like a cicada emerges from its shell (outlined in the Taoist scripture *Seven Bamboo Tablets of the Cloudy Satchel*, as cited in Needham, 1991, p. 141). The penultimate picture of the Ten Oxherding Pictures, the Reaching the Source, depicts how the selves disappear and the Sambhogakaya (retribution body) emerges (Trungpa, 2001). The *Past-and-Present Karma Sutra* (T. 3, pp. 620–653) tells the stories of the Buddha’s self-sacrifice out of great compassion (Skt. *mahā-karuṇā*) in past lives and the path of his awakening from prince to bodhisattva and finally to Buddhahood. The essence of great compassion and awakening is the “transformation of the basis of the mind” (Skt. *āśraya-parivrtti*) from the empirical self to transcendental Buddha-nature (Wangchuk, 2007, pp. 235–237), that is, the dialecticality of the subject. Derived from this dialectic, the holistic and dynamic nature of the self-displays compassion and the processes of change.

### 6.4. Emptiness (of self-nature)

Heidegger (1975) finds that one reaches transcendence by being projected into nothing: “Without the original manifest character of nothingness, there is no selfhood and no freedom” (p. 251). *Explanations of Diagram of the Ultimate* (Chinese: 太極圖說; *Taijitu Shuo*) of Zhou Dunyi (1017–1073) begins by declaring a basic presupposition: “from *wuji* emerges *taiji*.” *Wuji* symbolizes the undifferentiated nature of the mind—limitlessness, absoluteness, and nothingness. Correspondingly, the eighth of the Ten Oxherding Pictures unveils the primordial nature of the self by depicting an empty circle (Trungpa, 2001; Yamada, 2004). The Buddhist concept of non-self is based on the emptiness of self-nature (Skt. *svabhāva-śūnyatā*). According to the *Avataṃsaka*, everyone has infinite potential (analogous to the quantum superposition of all possibilities) and plays an active role in what nature manifests. The Buddha illuminates everything past and future in the present moment, demonstrating the emptiness of self-nature (*Avataṃsaka*, T. 9, p. 634a–b). Furthermore, according to the Madhyamaka philosophy, the Middle Way doctrine (comparable to Kant’s dialectics), the absolute truth manifests itself in the mutual implication of and sublation between the one and many (Murti and Venkatachala, 2008, p. 36). The Middle Way is based on the emptiness of self-nature, beyond the duality of nothingness and somethingness (Abe, 1985, p. 158). As the *Mahāprajñāpāramitā-sūtra* notes, “the great Bodhisattvas see the reality in everything, and the reality is neither one nor many” (T. 6, p. 676a, ll. 22–23). Thus, the emptiness of self-nature is defined by the fourth alternative of the fourth DMMS catuṣkoṭi, an awareness that there is neither the one (the first alternative) nor the many (the second alternative).

However, the most critical conceptual problem in the DMMS’ construction is the inherent incommensurability between different modes of cultivation. The transcendent God is the ultimate concern of pious Christians; by contrast, Mahāyāna Buddhists and Taoists aim to ultimately reveal the mind’s self-nature. The dialectical modeling of the DMMS suspends the researcher’s judgment and identifies a concept within the structure of its cultural system to reveal its essence, similar to Husserl’s transcendental-phenomenological reduction from non-reflective to reflective thinking.

## 7. Concluding remarks

This paper presents the first dialectical construction of an ontological model of self-cultivation, which frames the terminology in a tetralemmic dialectic that prevents concepts such as *yin*, *yang*, and self-nature from being limited to a subject-object dichotomy worldview. The DMMS unfolds the universal domains of self-cultivation and emphasizes that those cultural system domains dialectally coexist and imply each other. As the first step in Hwang’s epistemological strategy, its construction takes a dialectical approach that strategically regards spiritual development as the expansion or inward transcendence of the self-model to avoid controversy over different hierarchical rankings of spiritual development. Therefore, the DMMS is intended as a cross-cultural framework for further analyzing and integrating specific cultural systems in the second step of the strategy to construct additional culture-specific ontologies.

For example, to evaluate Buddhist practitioners’ advances in self-cultivation and help them develop a deeper awareness of their beliefs and paths, culture-specific ontological models can be further constructed by applying the DMMS framework to analyze and integrate the traditional models of self-cultivation in Buddhist classics, such as the Seven Stages of Purification in the *Visuddhimagga*, the Four Levels of Jhāna (meditation) described in the *Jhāna Sutta*, the Nine Levels of Meditation in the *Yogācārabhūmi-Śāstra*, and the Ten Oxherding Pictures in the Zen tradition (cf. Canda and Gomi, 2019), thereby creating subdomains to the DMMS. This study has analyzed each of the Ten Oxherding Pictures, showing how to integrate a traditional model of self-cultivation into the DMMS framework. The first and second pictures correspond to the first and second DMMS layers, respectively. The following four pictures are mapped onto the third layer of this framework, and the final four pictures onto the fourth layer. It is noteworthy that some stages of this traditional model are integrated into the DMMS framework as orderly and horizontally expanded domains in the same layer rather than as vertically ranked layers. Given the traditional model’s multiplicity of meanings, this hermeneutic approach is deliberately inclusive, enlightening, and exploratory rather than definitive. A culture-specific model reconstructed in this way is an improvement upon a traditional model, with an ontological commitment to the DMMS, and is more comprehensive and analyzable.

In constructing the DMMS, the catuṣkoṭi is employed to assimilate and integrate concepts and worldviews from various philosophies. It deeply probes different self-cultivation systems’ ontologies and merges them into a model to define such abstract concepts as emptiness and a non-self. This paper roughly outlines the Buddhist, Taoist, and Confucianist teachings about self-cultivation as examples to explain the DMMS and demonstrates how we can apply the DMMS to reveal and identify the 16 indispensable domains of a specific self-cultivation system. Concepts used in current scientific practices were considered in the development of the DMMS, and the definitions of the 16 domains are supported by philosophies or scientific theories. Thus, after modeling the ontology of self-cultivation, the related concepts may be scientifically treated.

The construction of the DMMS lends itself to three avenues for further research. First, it offers a broader perspective on spirituality, humanity, and reality than that offered by current mainstream psychological practices, without overemphasizing the self. The DMMS may be used by cultural psychologists as a map to determine whether they have overlooked a research domain. Second, indigenous psychologists could construct culture-inclusive theories based on the culture-specific ontological models created with the DMMS framework. Finally, future research must evaluate, refine, and improve the domain definitions of the DMMS by comparing more culture-specific ontologies of different cultural systems and achieving a fusion of horizons.

## Data availability statement

The original contributions presented in the study are included in the article/supplementary material, further inquiries can be directed to the corresponding author.

## Author contributions

The author confirms being the sole contributor of this work and has approved it for publication.

## Conflict of interest

The author declares that the research was conducted in the absence of any commercial or financial relationships that could be construed as a potential conflict of interest.

## Publisher’s note

All claims expressed in this article are solely those of the authors and do not necessarily represent those of their affiliated organizations, or those of the publisher, the editors and the reviewers. Any product that may be evaluated in this article, or claim that may be made by its manufacturer, is not guaranteed or endorsed by the publisher.
